# Distributed Optical Fiber Sensing Bonding Techniques Performance for Embedment inside Reinforced Concrete Structures

**DOI:** 10.3390/s20205788

**Published:** 2020-10-13

**Authors:** Mattia Francesco Bado, Joan R. Casas, Alinda Dey, Carlos Gil Berrocal

**Affiliations:** 1 Department of Civil and Environmental Engineering, Technical University of Catalonia (UPC), c/Jordi Girona 1-3, 08034 Barcelona, Spain; joan.ramon.casas@upc.edu; 2 Department of Reinforced Concrete Structures and Geotechnical Engineering (Vilnius Gediminas Technical University), Saulėtekio al. 11, 10221 Vilnius, Lithuania; alinda.dey@vgtu.lt; 3 Department of Architecture and Civil Engineering, Division of Structural Engineering (Chalmers University of Technology), Sven Hultins gata 6, 41258 Göteborg, Sweden; carlos.gil@chalmers.se

**Keywords:** distributed sensing, optical fibers, reinforced concrete, steel strains, structural health monitoring

## Abstract

Distributed optical fiber sensors (DOFS) are modern-day cutting-edge monitoring tools that are quickly acquiring relevance in structural health monitoring engineering. Their most ambitious use is embedded inside plain or reinforced concrete (RC) structures with the scope of comprehending their inner-workings and the functioning of the concrete-reinforcement interaction. Yet, multiple studies have shown that the bonding technique with which the DOFS are bonded to the reinforcement bars has a significant role on the quality of the extracted strain data. Whilst this influence has been studied for externally bonded DOFS, it has not been done for embedded ones. The present article is set on performing such study by monitoring the strain measurement quality as sampled by DOFS bonded to multiple rebars with different techniques and adhesives. These instrumented rebars are used to produce differently sized RC ties later tested in tension. The discussion of the test outputs highlights the quasi-optimal performance of a DOFS/rebar bonding technique consisting of incising a groove in the rebar, positioning the DOFS inside it, bonding it with cyanoacrylate and later adding a protective layer of silicone. The resulting data is mostly noise-free and anomalies-free, yet still presents a newly diagnosed hitch that needs addressing in future research.

## 1. Introduction

Structural health monitoring (SHM) is becoming an increasingly relevant branch of civil and structural engineering. Its key principles are the continuous assessment of newly built or pre-existent structures. This is translated into the monitoring of their serviceability health condition, risk mitigation, disaster prevention through well-organized and regularly updated maintenance [1] plans and, in the worst case scenarios, damage containment through quick emergency alarm. In order to prevent the adverse social, economic, environmental, and aesthetic impacts that may occur in case of structural and/or operational deficiency, it is important to have access to monitoring tools that encompass the features of reliability, quasi or entirely distributed measurement capacity, ease of application and cost-efficiency. The most commonly used sensors are electrical strain sensors, accelerometers, inclinometers, GPS based sensors, acoustic emission, wave propagation and more. Worth of mention are also in-situ triboluminescent optical fiber (ITOF) capable of yielding multifunctional smart structures with in-situ failure detection capabilities [2].

**Figure 1:**
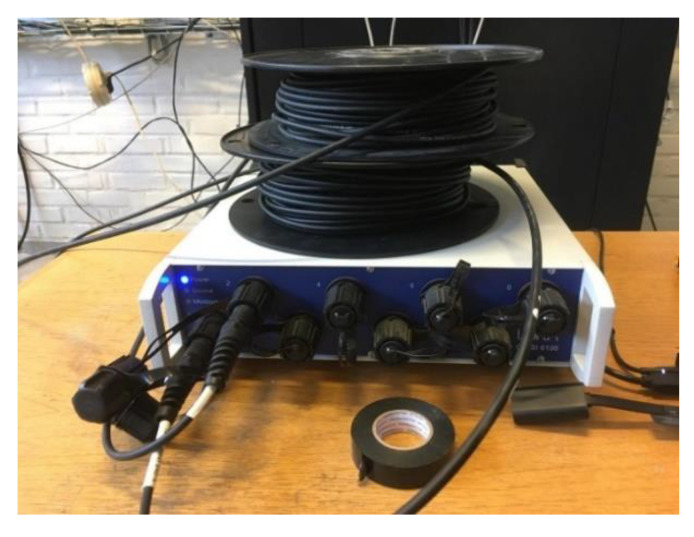
LUNA’s ODiSI 6000 Optical Backscatter Reflectometer.

Distributed optical fiber sensors (DOFS) are modern-day cutting-edge monitoring tools that are quickly acquiring relevance in the structural and civil engineering field [3]. These are very thin glass wires able to accurately measure strains (down to 1 µε), temperature and vibration in 2 or even 3 dimension [4]. This can be achieved in a completely-distributed manner (modern interrogation units can attain a spatial resolution of 0.63 mm) and with measurement frequencies of 250 Hz [5]. Thus, direct detection and characterization (including recognition, localization, and quantification or rating) of local strain changes generated by structural damage are intrinsic properties of such sensors. Furthermore, optical fiber sensors have some inherent advantages such as corrosion immunity, high durability, resistance to electromagnetic interference, small size and light weight that elevate them beyond the classic monitoring tools [6]. The functioning of these sensors is based on the fact that, assuming the characteristics of the light transmission within a fiber well known and the latter properly calibrated, any alteration (due to temperature and strain) are detected through back-scattered light by the optical backscatter reflectometer (OBR) (see Figure 1) and are finally translated to strain variation in each of the DOFS’ points [7].

**Figure 2:**
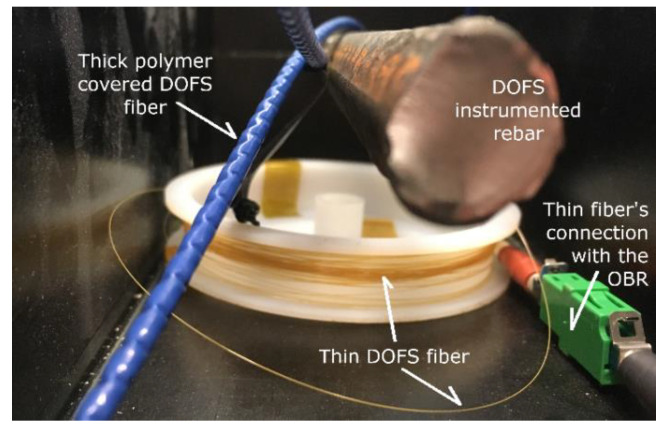
Thin polyamide cladded distributed optical fiber sensors (DOFS) (yellow) and thick polymer coated DOFS (blue).

The simplest DOFS sensor is a polyamide cladded glass wire which boasts a 125 μm diameter (see Figure 2). Multiple commercially available variations cover the latter with differently sized coating layers which span from rigid and deformed polymeric coatings (blue thick fiber in Figure 2) to thin fabric-like covers that consent a large amount of fiber winding.

The present work focuses on the thinnest form of DOFS which, despite its renowned fragility, it is also widely accepted as the most accurate of DOFS thanks to its direct bonding to the host material. Indeed, with the presence of a coating layer, the fibers’ measured results cannot be directly assumed to be equivalent to the substrate’s, because of the finite value of the coating’s shear modulus.

Embedding these sensors inside plain or reinforced concrete (RC) structures is also an increasingly popular trend in modern research, with the scope of comprehending their inner-workings and the physics of the bonding effects between concrete and steel. In most cases the DOFS are attached directly to the surface of the reinforcement-bars (rebars) before the latter are embedded inside concrete. Multiple studies [4,8,9,10,11] have demonstrated that the technique with which this bonding is achieved has a prominent role in the quality of the extracted data. Indeed, the highly sensitive nature of these hair-like fibers intrinsically exacerbates the probability of its malfunctioning and/or rupture during the concrete casting, maneuvering and testing phase. With this in mind, the optimal bonding technique is one that, not only ensures a continuous contact between DOFS and the structural surface to monitor (preventing their relative movement during the test), but also one that protects the former from accidental damage and testing-induced frictions with the concrete. Whilst thickly coated DOFS allow to bypass the issue completely, the strain reading precision is usually affected by the presence of the coating. In contrast, thinner coated DOFS, despite being more fragile, are much more effective in transferring strains thus leading to accurate readings that are much closer to the ones truly occurring in the RC structures. Yet again, its use demands abundant knowledge and acquaintance regarding the selection of the optimal bonding technique.

The importance of this issue has only lately been recognized as all the produced publications date back to last decade [12,13,14,15]. Worthy of mention, are two extensive studies analyzing the influence of different bonding adhesives on the strain output of polyamide cladded DOFS attached to bare rebars [11] and concrete surfaces [16]. Yet, no definitive comparative study has been developed for the case of DOFS bonded to rebars when embedded inside RC structures. The present work sets itself this exact goal.

Multiple publications display various manners of attaching the DOFS to the rebars (before their insertion inside concrete) and present diverse bonding techniques and adhesives. Their resulting DOFS strain profiles quality is varying and sometimes contradictory. The main parameter of such quality is the presence of strain reading anomalies (SRAs). These are localized, excessively large and inaccurate strain readings which do not present a direct physical explanation. Unfortunately, as will be visible later on, this is a very common and undesirable occurrence plaguing almost every single DOFS monitored campaign. Bado et al. [11,17] attempted to study these SRAs. According to them a strain peak at a general DOFS coordinate *x_k_* is usually considered as an abnormal reading if it presents a large difference with previous reading at coordinate *x_k−_*_1_. This can be both in terms of time (two consecutive strain measurements in the same DOFS coordinate) and space (two strain measurement performed in two neighboring DOFS coordinates at the same instance). Temporally speaking, unless a large load is suddenly applied on the DOFS-monitored structure, two consecutive measurements should present similar strain readings as the time interval between them (1/250th of a second), thus their load difference, is almost negligible. Spatially wise, if to consider DOFS tests with small spatial resolutions (0.63–1.3 mm), strains simply do not peak in neighboring sections without a certain amount of lead-up gradual growth [17]. Consequently, these anomalies can be considered DOFS reading errors or SRAs.

## 2. Literature Review

In order for the reader to both grasp the motivation of the present work and acquire the necessary groundings, it is important to first review the different experimental campaigns that have been developed embedding DOFS-instrumented rebars inside RC structures. The focus, here, are the used bonding techniques and their resulting strain reading quality (SRA presence). Hence, the following paragraphs constitute a concise collection of the aforementioned publications and its deducible conclusions represent the basis on the ground of which this work is developed. As visible later on, the review is subdivided in three general categories in which the bonding techniques can be subdivided, namely DOFS gluing without and with a protective layer and a groove incised in the rebar. Please note that, unless otherwise specified, the DOFS fiber used in the following experimental campaigns are constituted by a 9 μm diameter glass core (with uniform refractive index) covered with a 125 μm diameter polyamide cladding with no external jacket.

With the intention of comparing the DOFS performance when glued with cyanoacrylate and a two-component epoxy, Barrias et al. [8] bonded the fibers on the rebars of two small beams later tested under three-point loading. For both adhesives the extracted strains presented reasonably good agreement among each other and with embedded strain gauges despite a large amount of SRAs occurring as soon as the cracking starts. Nevertheless, the optical fiber glued with cyanoacrylate produced more consistent strain data whereas the epoxy-glued sensor showed slightly higher strain peaks.

Davis et al. [18] tested multiple 2.3 m long DOFS-monitored beams under a three-point bending setup. The polyamide cladded fibers were bonded with cyanoacrylate, more specifically Loctite 4851, resulting in very smooth strain curves outside the cracked section. On the other hand, some disruptive SRAs are present in the strain profiles of the beams subjected to corrosion damage.

Regier et al. [19] also used cyanoacrylate (Loctite 4851) to install a DOFS on the bottom rebar of a 1 m long RC beam tested under four point bending load. Though the measured strains demonstrated good agreement with the predicted strains for loads lower than 10 kN, for higher ones some large SRAs were spotted, specifically around the mid-span.

Malek et al. [20] compared polyimide and PVC (polyvinyl chloride) coated optical fibers both being glued to the beam’s reinforcement by means of a two-part epoxy (Loctite E-20HP). The authors found this adhesive more satisfactory with the polyimide coated fiber in terms of transferring strain to the core from its surroundings even in the deteriorated area.

Sieńko et al. [4] described a DOFS-monitored RC tensile members (ties) test where the fiber was glued to the degreased reinforcement bar along the longitudinal rib with an epoxy resin. The used fibers, though, differed from the current test’s due to the presence of a protective coating that consequently increased its diameter around seven folds (0.9 versus 0.125 mm) and led to a strain difference between the ones measured in the fiber and the one measured in the hosting steel.

Acknowledging the fragility of the polyamide DOFS, the scientific community started investigating a bonding technology that not only would warrant the correct bonding of the fiber to the structural surface but also a certain amount of protection from external factors. The most recurrent protection layer is a silicon-based one.

Davis et al. [10] proceeded to monitor the impact of (accelerated) corrosion on RC ties through DOFS as a supplement to visual inspections. The nylon and polymer cladded fibers were bonded with cyanoacrylate (Loctite 4851) and an extra silicone coating. The results, though, were very jagged strain profiles including a large number of SRAs.

Nurmi et al. [21] installed both nylon and polyamide cladded fibers on multiple rebars of an 80 mm thick slab with the aim of assessing the impact of the support conditions on its performance. Whilst both DOFS were bonded with cyanoacrylate (Loctite 4861), a silicon layer was positioned only on the polyamide fiber. Despite both fibers successfully sampling the structure’s strain behavior and its cracking pattern, some SRAs were present in the output data. The polyamide DOFS showed smoother strain curves, probably thanks to its silicone layer.

The use of a protective silicone layer was also commended by Brault and Hoult [22] after conducting an investigation on the potential of optical fibers as a strain measuring tool as well as its damage detection ability in differently sized beams. The authors further commended nylon cladded fibers compared to polyamide ones as the latter, despite the extra protection layer, displayed rougher strain curves with non-overlookable SRAs. Moreover, the former extrapolated measurements were in agreement with strain gauges’ output as well as the theoretical prediction.

Berrocal et al. [23] also used polyamide cladded fibers attached by a thin layer of cyanoacrylate further covered by an extra silicone protection. Their primary goal was to monitor the crack formation and evolution in beams subjected to monotonic and cyclic loading under a three-point bending setup. This bonding technologies allowed the successful detection of the microcracks width—even as miniscule as 40 µm. Furthermore, the authors endorsed this adhesive combination, as its strain results were in excellent agreement with the strain data from strain gauge sensors.

**Figure 3:**
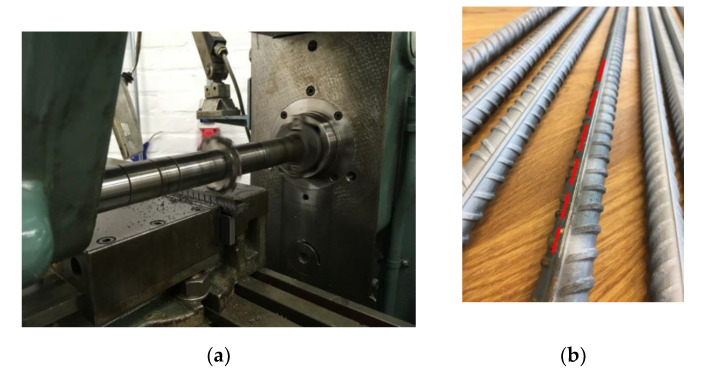
(**a**) Milling of the groove along the rebar’s longitudinal rib and its (**b**) resulting incision marked with a dashed red line.

A rather novel and advanced DOFS protection technique establishes the incision of a groove on a rebar’s surface (usually on the longitudinal rib as in Figure 3) which later welcomes the DOFS inside it. As will be visible in the following publications, this is believed to provide further protection compared to simply gluing the fiber to the rebar’s surface (Quiertant et al. [24]). Yet, it is quite surprising that, despite this technique’s non-negligible financial and time costs, a comprehensive study on the potential improvement that it brings to the table (in terms of DOFS reading quality) has yet to be performed. The present paper intends to perform such analysis.

The efficiency of this arrangement was commended by Michou et al. [25] who used a 1 × 1 mm groove incised along the rebar of a 1.15 m long RC tie in order to study the evolution of the concrete cracking, its transfer lengths and tension stiffening. Despite no specifications are given on the kind of adhesive used, the mere presence of the groove led to an output characterized by smooth curves and easily recognizable strain peaks indicative of cracks.

Haefliger et al. [26] used this technique too, engraving a longitudinal section of 1 × 1 mm along the rebar and later gluing the optical fiber inside it with epoxy. The bars were positioned inside a 600 × 600 mm, 125 mm thick RC square panel subjected to diagonal tension in a universal testing machine (UTM). This technique resulted in notably smooth strain curves. The optical fiber diagnosed the correct location of the cracks which were further validated by digital image correlation (DIC). Yet, the strains in the cracked sections within the range of the transition zone were lower than expected.

Bado et al. [27] carried out a DOFS monitoring tensile tests on different RC ties by positioning the DOFS inside a groove milled on the bar’s surface and later glued with cyanoacrylate. Here, the extracted strain profiles’ peaks, correctly indicated the crack locations (further corroborated by a DIC monitoring) but a significant number of strain reading anomalies (SRAs) were found.

The groove-cyanoacrylate combination was further used in Bado et al. [17] with similar results. Indeed, the strain profiles extracted from two more RC ties presented a consistent amount of SRAs which were later removed through a newly introduced post processing algorithm.

In their research Bassil et al. [28] studied the output of a DOFS monitoring campaign (bonded with a two component epoxy and positioned inside a groove) inside a 1 m long RC beam subjected to a three point loading setup. The authors witnessed noticeable SRAs immediately after micro-cracks began forming around the rebars, steeply increasing in magnitude with every load increment.

The following conclusions can be extracted from the above literature review:

- The friction between DOFS and the surrounding concrete is the main reason for the appearance of SRAs. Therefore, the bonding techniques that seems to best insulates the former from the latter seem to yield to the most SRA-free outputs.
- Bonding adhesives aside, three main DOFS/rebar bonding techniques have been adopted up to now:
  ○ Simply gluing the DOFS on the rebar’s surface away from the transversal ribs;
  ○ Gluing the DOFS to the rebar’s surface and covering it with a silicone-based protection layer;
  ○ Incising a groove on the rebar’s surface and positioning the DOFS inside it before gluing it;
- The presence of silicone and of a groove seems to improve the quality of the DOFS extracted profiles;
- Despite such improvement, none of these techniques seems to unequivocally guarantee the absence of SRAs;
- A combination of groove and silicone has not been tested yet.

As previously mentioned, a deep study on the performance of each of these techniques has yet to be developed as, all we have at the present moment, is essentially a conglomeration of scattered data from different experimental campaigns with different execution conditions. To tackle this issue, the present paper will display the results of a single DOFS-monitored experimental campaign on multiple RC ties which include all the above mentioned DOFS/rebar bonding techniques. A strain reading quality analysis will follow up in order to extract conclusions on which is the most performant one.

## 3. Experimental Setup

### 3.1. RC Specimen Description

**Figure 4:**
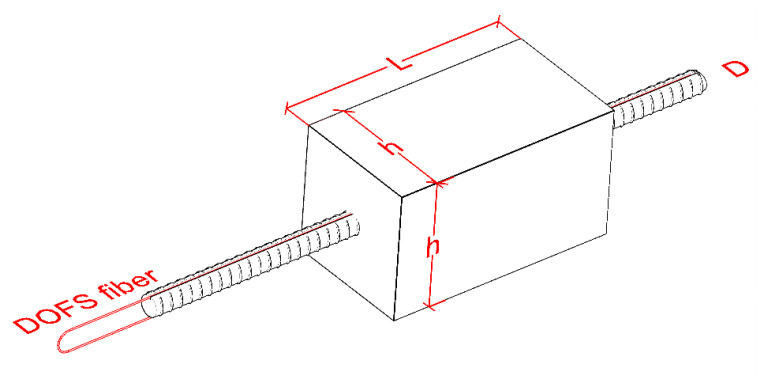
Reinforced concrete (RC) ties’ general geometrical dimensions.

The present paper displays the results of an experimental campaign developed on multiple squared RC ties with DOFS-instrumented rebars. Each of the RC members will be indicated with a code such as 15D20_24 that should be interpreted in combination with Figure 4, which provides indications on the geometrical features of each RC member.

The first digits of the code are representative of the concrete prism’s cross sectional size (h in Figure 4), the digits that follow the letter D are representative of the diameter of the rebars (D in Figure 4) whilst the digits following the underscore symbol (_) are representative of the length of the concrete prism (L in Figure 4). The geometrical design of these RC members satisfies the conditions necessary to perform a double pullout test-powered study [29] on the influence of the longitudinal dimensions of a RC tie on the bond stress/slip present in the interface between rebar and concrete; topic of future publications.

For the abovementioned example code, the member in question would be a 15 × 15 × 24 cm concrete prism pierced by a Ø20 rebar. From Figure 4 it is possible to see how the rebars are monitored on both of their sides through a single DOFS twisted backwards whenever the latter runs outside the concrete prism. As visible later on, two members were monitored by a single fiber run due to its original short extension whilst another one has three DOFS runs (two on one side and one on the opposite). The key advantage of monitoring multiple times the same rebar with a single DOFS is the possibility of using multiple bonding techniques inside a single member (to verify their performance). Thus, the uncertainties connected to the potential difference among different samples is removed.

**Figure 5:**
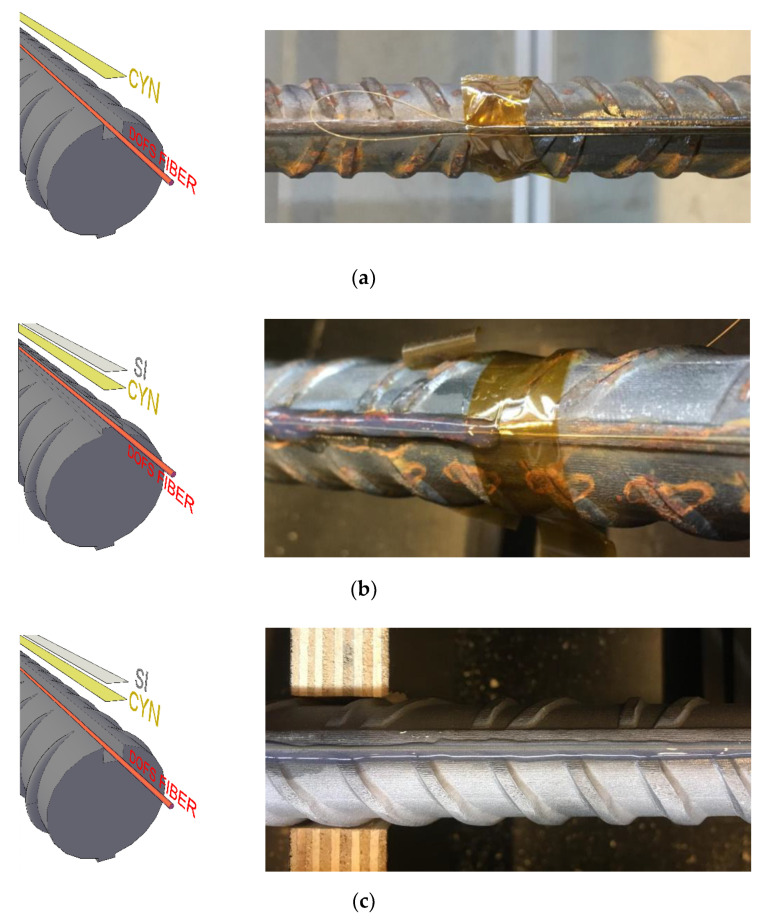
Graphical and photographical representation of the three tested bonding techniques (**a**) positioning of DOFS in an incised groove and bonding it with CYN (**b**) bonding it with CYN with an additional protective layer of SI (**c**) positioning of the DOFS in a groove, bonding through CYN and protection through SI.

The bonding technologies in use, illustrated in Figure 5, are the following ones:

1. DOFS positioned inside a groove and bonded with cyanoacrylate (CYN) as visible in Figure 5a;
2. DOFS glued to the rebar’s surface with CYN with a protective layer of silicone (SI) on top, as visible in Figure 5b;
3. DOFS positioned inside a groove, bonded with CYN and further covered with SI. As visible in Figure 5c.

It is worth mentioning that, in Figure 5c, the groove is hardly visible due to its small size (as evident in Figure 3b) and color similarity with the background. Additionally, hereafter rebars with an incised groove will be referred to as (w/groove) whilst the ones without it as (w/o groove).

The concrete’s mean compressive strength was equal to $f_{cm}=47.89\;\mathrm{MPa}$, its modulus of elasticity to $E_{cm}=28,159\;\mathrm{MPa}$ and finally its tensile strength to $f_{ct}=2.89\;\mathrm{MPa}$; all of them measured at 28 days. The steel rebars were instead characterized by a yielding strength of $f_{ym}=533.31\;\mathrm{MPa}$.

### 3.2. The Experimental Campaign: The Concept and Its Phases

In order to assess the quality of a DOFS/rebar bonding technique multiple aspects should be taken into consideration. As seen, first among these, is the cleanliness of the extracted signal (deeply related to the amount of SRAs) but also the adhesives’ resistance to the external forces that would otherwise separate the sensor from its surface.

Finally, it is a crucial requirement that the bonding technique does not influence the strains being read by the DOFS. It should be guaranteed, to the maximum extent possible, that what is being read are the actual strains occurring in the monitored surface rather than a strain present solely in the fiber.

In order to tackle this last key point, the experimental campaign is subdivided in two distinct phases. The first one will make use of DOFS-instrumented bare rebars whilst the second will test these same rebars this time embedded inside concrete prisms.

**Figure 6:**
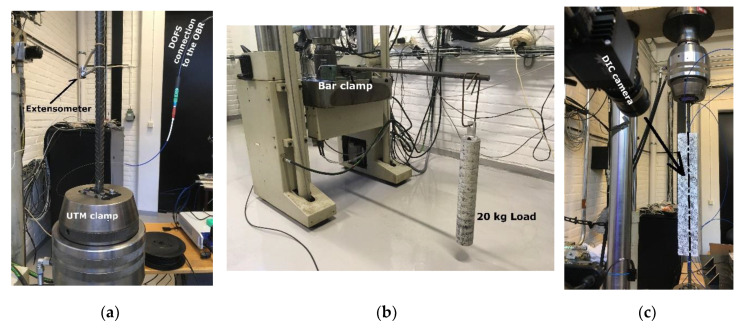
Test setup for the (**a**) universal testing machine (UTM)-assisted tensile test on a bare rebar (**b**) bending test (**c**) UTM and digital image correlation (DIC)-assisted tensile test on a RC tie.

This first phase, is aimed at assessing the potential influence that the presence of a layer of SI or a groove might have on the extrapolated DOFS readings when no concrete is involved (bare rebar). This is assessed both in tension and in bending. The first is achieved by placing the rebar in a Universal Testing Machine (UTM), as in Figure 6a (with an extensometer gauge for comparative purposes) two times. Both times three load cycles were performed, each reaching a third of the yielding load. The first time the rebar is tensed with such program, the DOFS is simply glued with CYN whilst the second time it is further covered by a layer of SI. The performed measurements are later compared in an attempt to spot any potential difference caused by the extra layer. These, obviously, need to be larger than the slight but expected difference induced by the impossibility of repeating the same exact clamping conditions. The tested rebars are two, one w/groove and one w/o groove with the express purpose of comparing their results to study the influence of the groove.

The bending test is instead achieved by clamping one extremity of the rebars and applying on the opposite one (at a fixed distance) a 20 kg load (Figure 6b) in order to create a bending stress and a clearly distinguishable strain profile. Once again, this process is done twice in order to monitor the instrumented rebars strains with a CYN-bonded DOFS and later a CYN+SI bonded DOFS.

The second phase is aimed at studying the DOFS/rebar bonding techniques performance (in terms of quality of the extracted readings) when the latter is embedded inside a concrete prism. Therefore, it includes the embedding of the instrumented rebars inside concrete forming RC ties and later their testing by means of the UTM (as visible in Figure 6c). This last test is integrated with a DIC monitoring of the RC ties’ surfaces aimed at studying the crack formation, evolution and relation with the interiorly sampled rebar strains. The loading program is a simple displacement-controlled monotonic tensile load increased at a speed of 1.5 mm/min until yielding of the rebar.

## 4. Test Results

### 4.1. Phase 1: Bare Rebar Testing in Tension and Bending

**Figure 7:**
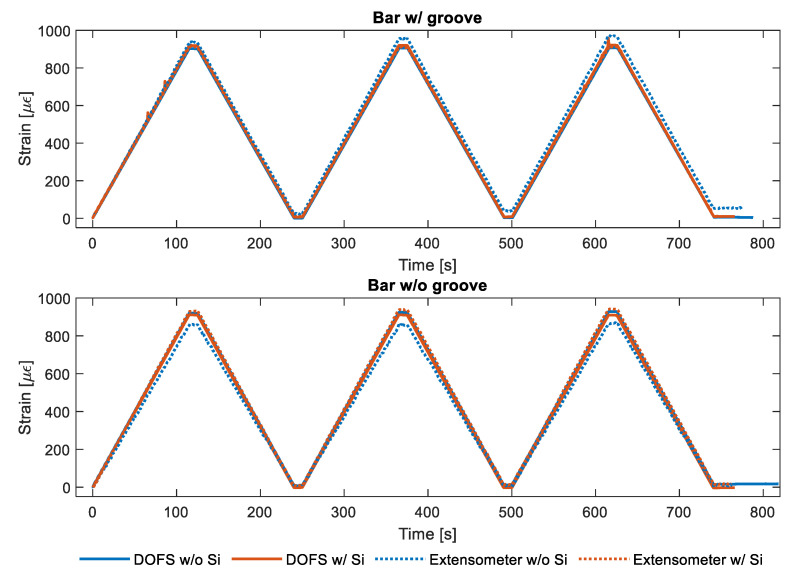
DOFS and extensometer output readings of the tensile test.

**Figure 8:**
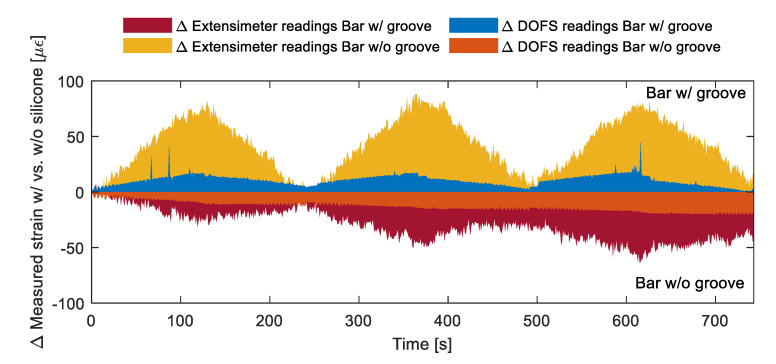
Bar w/groove and w/o groove’s strain reading differences when DOFS + cyanoacrylate (CYN) are or are not covered by silicone (SI).

Figure 7 plots the strain measurements performed by the DOFS and by the extensometers connected to the two rebars w/groove and w/o groove. As visible both graphs include two pair of readings, one (in blue) representative of the strains recorded by the DOFS glued with CYN and the other (in red) representative of the strains recorded by the same DOFS glued with CYN and covered with SI. An excellent agreement is present between the DOFS strain curves of both graphs indicative of the small influence that a layer of SI exercises on the tensile readings of a bare bar. Such agreement outshines the ones of the classic extensometer. Moreover, for the case of the bar w/groove, the extensometer even fails to detect that the zeroing of the applied load at the test’s end whilst DOFS actually does. The extensometer’s lower readings quality is definitely a function of how/where the tool is attached on the rebar compared to its transversal ribs but, then again, it is an uncertainty that is entirely surpassed by DOFS. Figure 8 plots in more detail the strain reading differences of the two rebars when SI is present and when it is not. As DOFS’ does not surpass 15 με in either bar (an amount possibly induced simply by a variation of the grip position), it can be safely stated that in tension the presence of SI does not influence the strain readings and thus it is safe to apply it for further protection of the fiber. For the extensometer, instead, the reading variations are expectedly higher for both bars suggesting, once again, the greater reliability of DOFS. Finally, as can be seen in Figure 7, the presence of a groove also does not seem to alter the strain readings suggesting that it too, in tension, can be inserted sans souci as an extra protection for the fiber.

**Figure 9:**
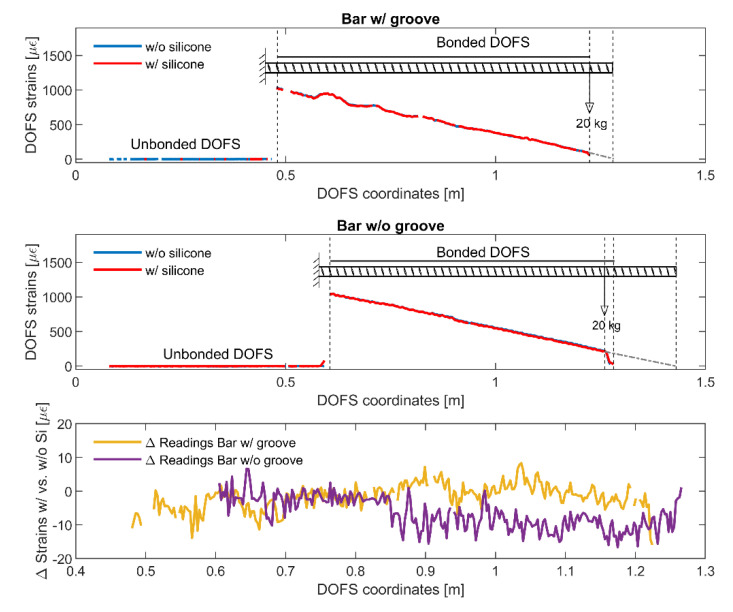
Strain profiles of the rebars (w/and w/o groove) under bending and the difference of values w/and w/o SI.

Moving on to the bending test, Figure 9 plots the DOFS strain measured in absence (in blue) and in presence of a SI protection layer for both bars w/and w/o groove. Due to the difference of DOFS bonding length in the two rebars, an illustration of the latter is inserted in both plots in order to help the reader better comprehend the shape of strain profiles.

It should be kept in mind that the initial segment of the graph, oscillating around the value of 0 με, is simply denoting the strains inside the un-bonded and loose section of the DOFS. Only once the first DOFS section of the fiber is bonded, then the profile jumps upwards reporting the rebar’s highest strain. Once again, the presence of silicone is hardly perceptible, as confirmed by Figure 9’s bottom graph, leading to quasi-identical strain profiles. Once again, the largest recorded strain measurement difference between SI covered bars and not is around 15 με for both rebars. The presence of the groove too, hardly influences the DOFS readings. Indeed, the difference between the rebar w/and w/o groove is around 10 με thus completely justifying the use of a groove for warranting extra protection to the DOFS.

The unequivocal conclusion of the first sub-section is that neither the addition of an extra protective layer of SI nor the positioning of the DOFS inside a groove incised along a rebar influences the DOFS strain readings when the rebar is bare and tested in tension and bending. Therefore, if the test’s seconds phase (RC ties) will display reading alterations, they can safely be attributed to the concrete surrounding the instrumented rebar and its friction with the latter.

### 4.2. Phase 2: RC Ties Testing

The present sub-section is further divided into two parts. The first is interested in studying the performance of various DOFS/rebar bonding techniques whenever the instrumented bar is embedded inside concrete without the possible interferences brought on by the latter cracking. The second instead, embraces exactly this; it being a crucial part of any RC structure testing.

#### 4.2.1. Non-Cracking RC Ties

**Figure 10:**
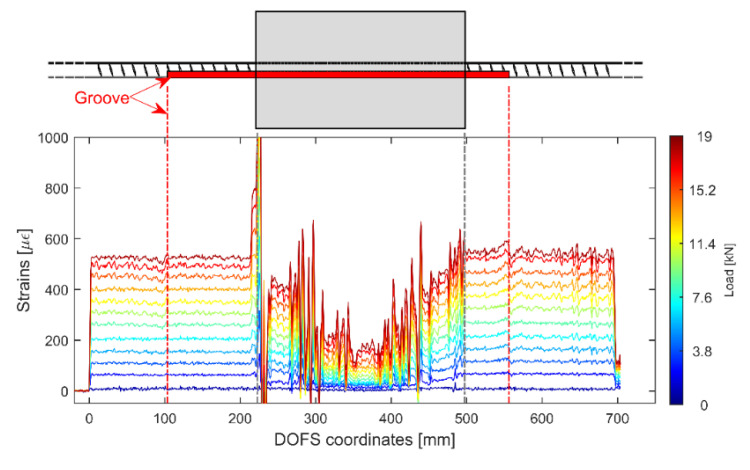
Strain profiles of a 15D20_27 RC tie test with the DOFS bonded with a combination of groove and CYN.

The first tested RC tie was a 15D20_27 member with its DOFS positioned in a groove and bonded with strictly CYN (no protection layer was placed). The test was intended to substantiate whether the mere presence of the groove could guarantee sufficient protection to the DOFS without requiring an extra protective layer. Figure 10 displays some of the strain profiles extracted from the aforementioned test along with an illustration of the member’s geometry in order to properly correlate the plot data with the actual physical surface/surrounding in which the DOFS is located. As visible, the profiles’ sections corresponding to the segment of the instrumented rebar embedded inside the concrete is full of SRAs (induced by the concrete’s friction on the unprotected DOFS) making the actual profile barely readable. This is in line with multiple results reported in the literature review, thus leading to the conclusion that this technique most definitely is not recommended for this kind of DOFS-monitored testing. Yet, it should be mentioned that a possible influencing parameter could be the actual depth of the groove (quite shallow in the present test). It is possible that the deeper it is the better protection it provides to the rebar.

Then again one of the greatest advantages of DOFS is its quasi-disregardable intrusiveness which is in strong contrast with the idea of having to incise a deep groove in order to ensure its correct functioning. Furthermore, for smaller rebar diameters such as Ø8 a deep incision is inadmissible as it could significantly alter its mechanical behavior.

**Figure 11:**
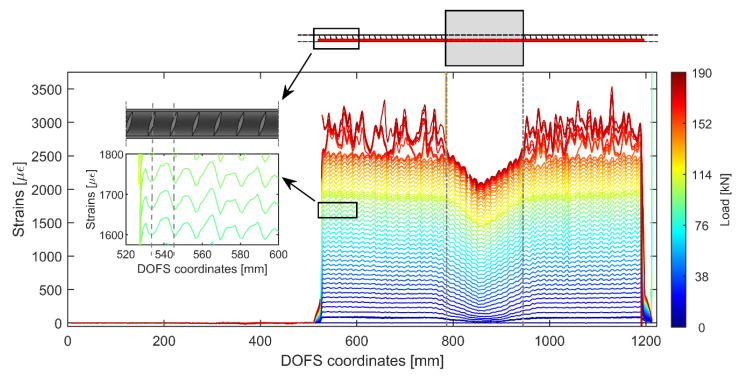
Strain profiles of a 15D20_21 RC tie test with the DOFS bonded with a combination of groove, CYN and SI.

Figure 11 displays the strain profiles of a 15D20_21 RC tie where the DOFS is first positioned in a groove and then bonded with CYN and SI.

As can be seen in Figure 11 the profiles are much better than Figure 10’s as a testament to the higher performance of its bonding technique. Indeed, hardly any SRAs are visible and the strains are monitored all the way to the yielding load (around 167.5 kN) and beyond. The peaks present at the higher loads are not SRAs (as was the case in Figure 10) but rather the physical representation of the rebar’s different sections slowly plasticizing one after the other and therefore giving way to drastically higher strains than the non-plasticizing sections.

**Figure 12:**
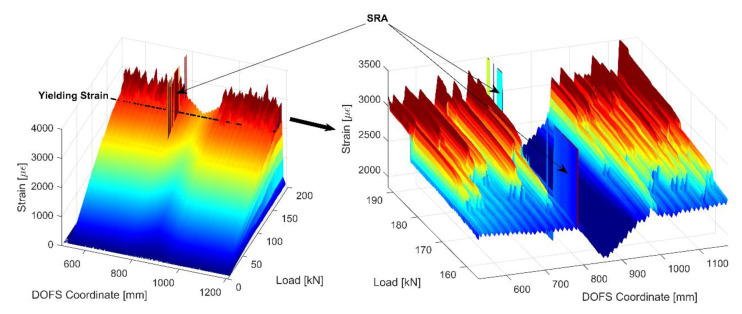
Three-dimensional representation of the strain evolution of member 15D20_21 with a zoom of the higher strains showing the progressive yielding of the different cross-sections of the rebar.

Figure 12 provides a three-dimensional representation of member 15D20_21’s strains displaying a smooth increase up until the yielding strain $\varepsilon_{sy}$ beyond which it is visible how little by little all the rebar’s cross sections progressively plasticize (indicated by a sudden peaking of the strains).

This by itself is a significant step forward from many literature’s DOFS embedded tests which stop producing reliable results at loading stages numerous folds inferior to the present one. This is, furthermore, very encouraging when it comes to the DOFS/rebar bonding technique performance. 

**Figure 13:**
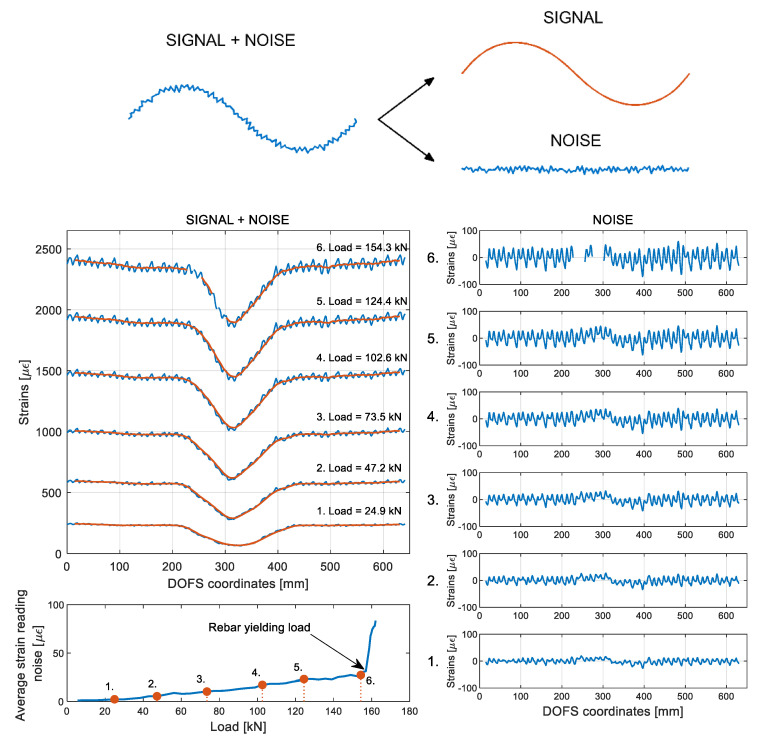
Distinction of signal and noise for an example curve (top) and evolution of the rib-induced noise with the load of member 15D20_21.

Moving back to Figure 11, a small zoom is provided on its left of the rebar’s strains profile outside of the concrete as reported by DOFS. An oscillation of the profile is visible which could easily be confused with signal noise. Yet, it is not noise but rather the influence of the transversal rib on the performance of the rebar. As a matter of fact, whenever a rib is present, the cross-section of the bar is automatically larger therefore the carried stress (and consequent steel strains) is inferior. Oppositely, whenever the rib is absent the carried stress is higher. The reiteration of this pattern leads to the abovementioned oscillating profiles. Yet, unless a researcher is interested specifically in the rib’s influence on the bar’s performance, the latter can be considered signal noise as the useful information signal that is usually pursued is strictly the global profile of the strain curve. As will be visible later on, this particular noise is also a function of the bonding technique in use, thus it is automatically elected as a study parameter for establishing the most performant bonding technique. In order to separate the noise from its original signal (as in Figure 13) a Fourier filtering solution can be used, thus relating a signal sampled in time or space to the same signal sampled in frequency, or a moving-average filter, thus sliding a window of customizable length along the data and computing the averages of the data contained in each window. For the scope of the current work the latter is deemed to be sufficiently performant, hence it is the used methodology.

What is also noticeable in Figure 11, is that the magnitude of such newly defined rib-induced noise becomes increasingly larger with the load thus increasingly disturbing the sought-after signal. Figure 13 shows its occurrence for member 15D20_21’s six study case stages along its test. The latest of these stages has average noise peaks of height 27.5 με which, considering its corresponding load of 154 kN, is still very reasonable. As per the bottom graph of Figure 13, the average strain reading noise seems to be increasing at a regular pace with the load suggesting a correlation between the two.

This was expected since, being the oscillations caused by the presence of the transverse ribs, the absorbed stress taken by the latter should also increase linearly with the load while in the elastic range. The only moment when the latter is broken is when the steel’s yielding stress is reached. Beyond this point, as seen in Figure 12, large peaks start forming wherever the rebar’s cross-sections start yielding.

**Figure 14:**
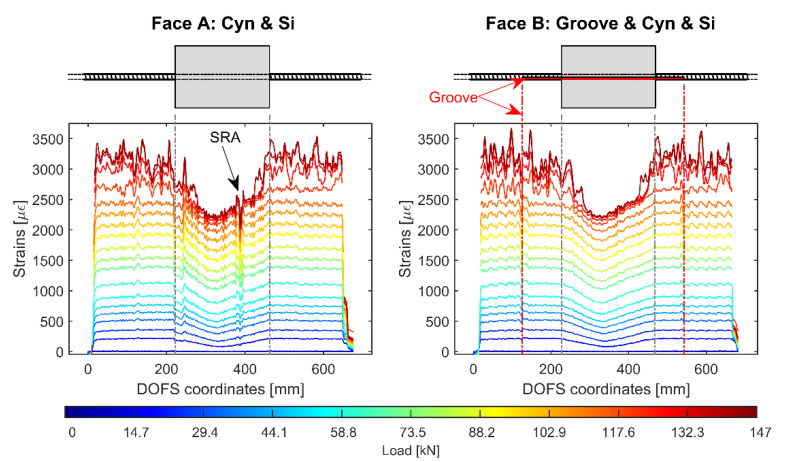
Strain profiles of 15D20_24_1 RC tie test with the DOFS bonded on both sides of the rebar by means of CYN and SI on one side (Face A—**left**) and a groove + CYN + SI on the other (Face B—**right**).

Moving on to the following RC tie, Figure 14 displays the DOFS strain readings of the first of two 15D20_24 members (henceforth referred to as 15D20_24_1) with its DOFS being bonded on its opposite faces. One face (henceforth referred to as Face A) uses CYN and SI whilst the opposite (Face B) adds a groove. The latter, though, is not as long as the rebar itself (see Figure 14’s Face B RC tie illustration) therefore allowing to check, on a single face, the DOFS reading performance of two separate bonding techniques. What can be quickly spotted is the larger amount of noise that characterizes Face B’s readings when performed outside of the groove versus inside it. Furthermore, along with a higher noise level of the signal, Face A’s plots seem to include more SRAs. This is potentially due to the lack of a protective groove on its side.

**Figure 15:**
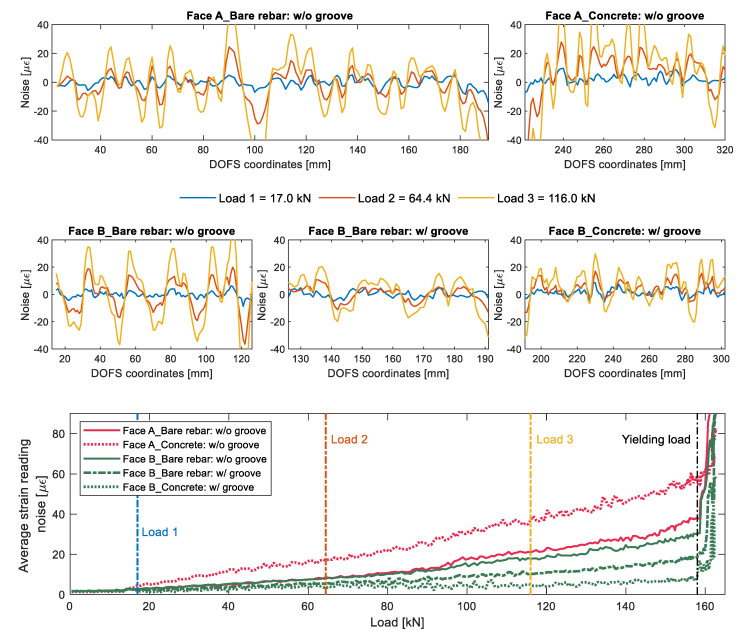
Rib-induced noise analysis of the outputs of member’s 15D20_24’s test (top five subplots) and of their relative average reading noises (bottom subplot).

Figure 15 dwells deeper in the DOFS’ signal noise, displaying its values under three load levels. A discrete noise magnitude difference is noticeable according to the difference of DOFS/rebar bonding technique and according to the presence or absence of concrete. Figure 15’s lower plot summarizes the increasing noise magnitude as a function of the load.

Both faces of the rebar report similar high noise levels whenever the DOFS is bonded outside of the groove whilst lower levels are consistently reported (both outside and inside the concrete) whenever the DOFS is positioned inside it. Once again it seems that the presence of the groove translates in an improved signal quality both from a noise and anomalistic point of view.

What is also on view is that, just like in Figure 13, the noise magnitude increases linearly up until the yielding load. Therefore, henceforward the noise level magnitude can be studied simply by means of a fitted straight line’s slope coefficient. For example, Face A’s versus Face B’s average strain reading noise slope coefficient when inside the concrete is 0.3636 versus 0.0360, indicating the latter’s higher quality. Now that the basic concepts of the rib-induced noise and its way of quantifying it have been introduced, the other members’ analysis will be performed jointly at the end of this section.

**Figure 16:**
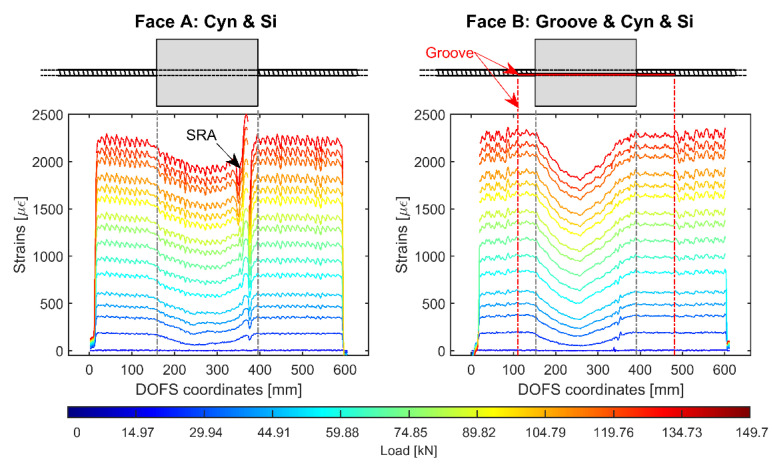
Strain profiles of 15D20_24_2 RC tie test with the DOFS bonded on both sides of the rebar by means of CYN and SI on one side (Face A—**left**) and a groove + CYN + SI on the other (Face B—**right**).

Figure 16 displays the DOFS strain measurement of an identical member, 15D20_24_2, with also identical bonding techniques.

The reason behind the testing of such member was the idea of attempting the replication of the previous member’s results in order to consolidate them. While most of the above conclusions still apply to the present member (the presence of the groove providing the least noisy and anomalistic results both inside and outside the concrete), an unforeseen phenomenon also came up. Indeed, whilst in Figure 14 both faces provide similarly shaped plots, Figure 16’s are quite different. Face A’s profile inside the concrete loses the central pointy tip (expected in RC tie tests) and appears more curvilinear (instead of the expected pair of crossing linear profiles). Such difference can be attributed to a non-overlookable bending of the RC tie during the test, induced by a lack of straightness of the bar itself. Unfortunately, discrete amounts of bending occur in numerous RC tie tests due to the impossibility of having perfectly straight rebars. Whilst this is an accountable phenomenon, the effect of such bending on the DOFS (or on the adhesives bonding it) is unseen and deserving of further studies. A possible explanation to Face A’s profiles could lay in the additional stretching that the bending-induced-tension of the concrete communicates to the adhesives (especially on the very deformable silicone) and thus to the fiber. Additionally, further studying should also be dedicated to assessing the effect of bending-induced-compression. This is in line with other researchers’ results [11,16] that suggest a certain “strain relaxing” and ”strain redistributing” influence of the silicone when it is used as a DOFS protecting material. Unfortunately, this phenomenon strongly influences the recommendability of the present bonding technique for this particular use. Furthermore, it should be kept in mind that such alteration is truly revealed only when monitoring multiple facets of the same rebar. Indeed, if only a single DOFS deployment is performed, all that is extracted from the test is such altered data is with no way of assessing its correctness.

Now that all the non-cracking members’ DOFS readings have been presented, Table 1 summarizes all their rib-induced noise by means of their slope coefficient in an attempt to establish which bonding technique produces the least amount.

It should be mentioned that for member 15D20_27 it is nonsensical to calculate the amount of noise when the full graph is predominantly SRAs, therefore an ∞ noise value is given. Table 1 clearly indicates the groove combined with a protective SI layer as the bonding technology that leads to the smallest rib-induced noise growth with load ensuring the clearest DOFS signal even at the end of the tensile test.

#### 4.2.2. Cracking RC Ties

**Figure 17:**
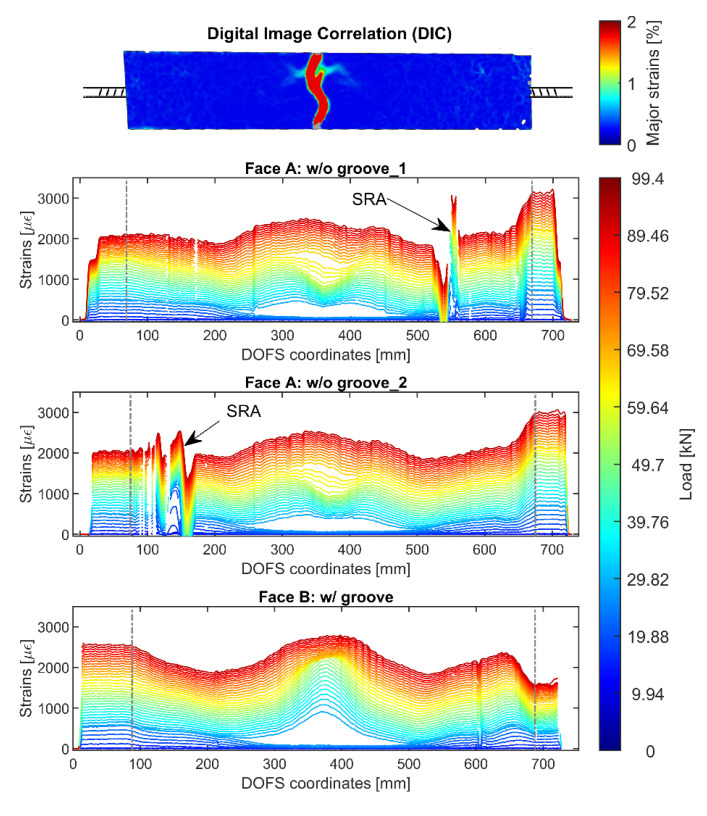
DOFS extracted profiles of member 12D16_60 as monitored by three runs of the same fiber, two on Face A and one on Face B.

The first RC cracking tie under scrutiny is 12D16_60. The member has the peculiarity of being monitored by three runs of the same fiber (possible thanks to its extended length) achieved through a simple twist, as shown in Figure 5a. Two DOFS runs are performed on Face A, where both bondings are achieved with CYN and SI w/o groove, and one on Face B w/groove (with CYN and SI). Their outputs are plotted in Figure 17 which includes a DIC illustration (on top) of the position of the crack in order to correlate it to the member’s rebar strains.

Despite a relevant amount of bending is evident in each of three plots, a clear variation of the strain profile can be noticed in correspondence to the formed crack. Indeed, this variation takes the form of a single (Face B) or double pointed (Face A) peak whose axes coincides with the crack displayed in the DIC. Multiple SRAs can be seen in both of Face A’s plots whilst only a small un-intrusive one can be spotted in Face B’s suggesting the superiority of the latter’s bonding technique. In line with what was concluded in the previous sub-section, also the noise produced by the combination of groove, CYN and SI is inferior to the bonding technique using just the last two elements.

**Figure 18:**
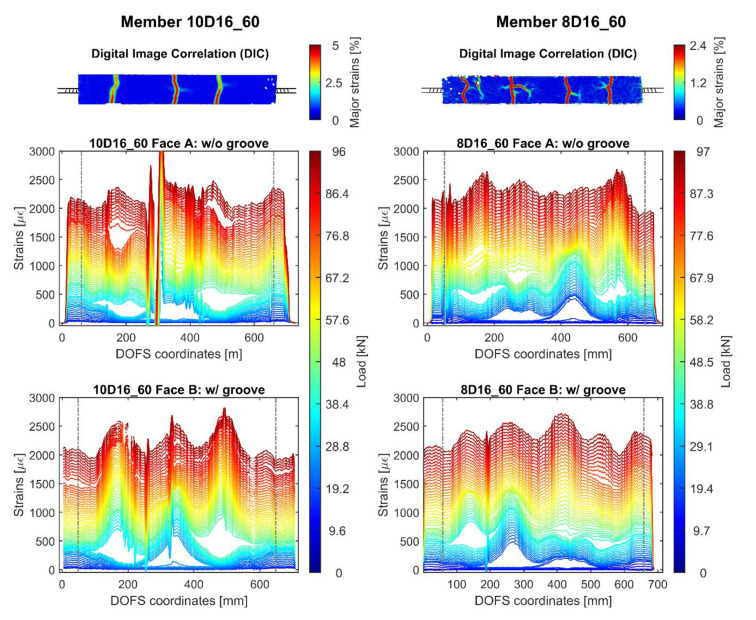
Member 10D16_60 (**left**) and 8D16_60’s (**right**) rebar DOFS strain profiles for the latter’s opposite faces, each welcoming different bonding techniques, and their DIC monitoring.

Figure 18 expands on the previous one showing the DOFS and DIC outputs of members 10D16_60 and 8D16_60. Just like before, the members’ Faces A use simply CYN and SI whilst Face B adds a groove to the mix. Similar observations to the previous figure’s can be performed. Indeed, strain peaks correspond well to DIC’s cracks location and the signal noise level is inferior for DOFS positioned inside a groove versus outside one. Interestingly, though, member 10D16_60 displays a consistent amount of SRAs in both faces demonstrating that even the combination of groove, CYN and SI is not completely fail-proof.

Ideally, the steel rebar’s strains in the cracked section (where concrete is absent) should be equivalent to the steel strains present on the bare rebar outside of the concrete prism. Yet, due to the presence of bending in the rebar, this assumption cannot be made. Furthermore, member 10D16_60’s two plots display two different crack-induced peak heights, Face A’s smaller than Face B’s. As mentioned above, it is only through a double DOFS monitoring of member’s 10D16_60 rebar that we are able to diagnose this difference. If only Face B had been monitored, its profile would automatically be considered representative of the rebar’s strain state. With this in mind, extreme care should be adopted whenever extracting and reporting such results.

Member’s 10D16_60 crack-induced strain peak height difference between Faces A and B, could potentially be attributed to the two following issues; the above mentioned extra-stretching communicated to the DOFS by the bending-induced-tension inside the concrete and/or to the under-performance of SI which fails to properly isolate the DOFS from the concrete’s pull whenever the latter’s cracks open. Differently so, this issue was less pronounced for both fibers deployed in member 8D16_60 probably due to either a positioning of the DOFS half way between the bending-induced tensile and compressive area or to a thicker and more protective layer of SI. Further research is necessary to comprehend this phenomenon as no sufficient data is available at the present moment.

Nevertheless, three potential ways of preventing this phenomenon from happening are:

- A preparatory study of the rebars’ minute bending (could be achieved through 3D scanning technique for example) and the bonding of the DOFS strictly in those areas where the straightening of rebar will cause compression;
- Use of a different protective agent than SI;
- Application of a thicker layer of SI than the one applied to further insulate the DOFS.

The following section will finally draw some conclusions on the SRA-preventing power of each DOFS/rebar bonding technique, accounting both cracking and not-cracking RC ties.

## 5. SRA Analysis per Every DOFS/Rebar Bonding Technique

**Figure 19:**
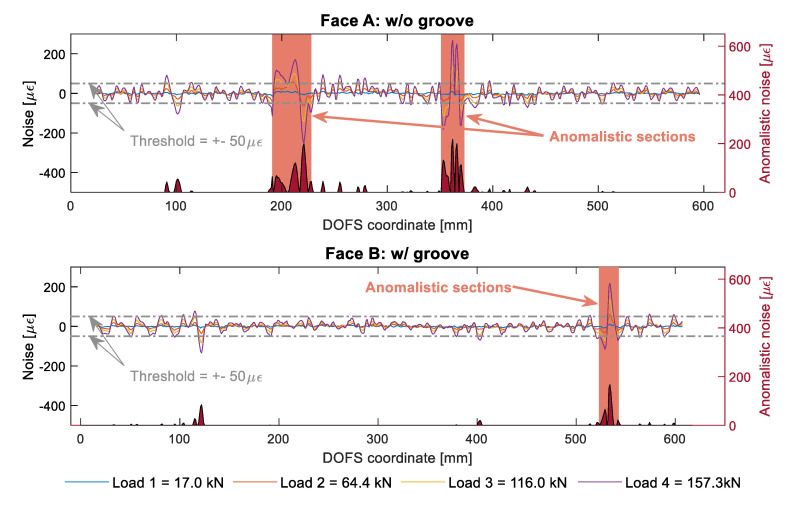
Member 15D20_24_2 Face A’s (**top**) and B’s (**bottom**) noise level study for SRA analysis.

In order to properly diagnose an SRA and clearly distinguish it from the signal’s noise, a unique method is here presented. Taking as example member 15D20_24_2, Figure 19 analyses its DOFS signal noise level for four distinct loads.

For clarity purposes, being the noise increasingly larger with the load, only the latter will be studied on the lookout for SRAs. Then, if to assume a customizable threshold of ±50 με, any noise level higher than the latter can be considered anomalistic. The selected threshold may vary according to the researchers’ objectives, desired strictness and subjective sensibility on what should be labeled as anomalistic. Figure 19 adds an extra axis to each of the graphs where the amount of this newly-labeled “anomalistic noise” is represented providing a graphical representation of the location and gravity of the anomalies. Some anomalistic sections can be detected in both faces (despite the largest amount being on Face A) where most of the SRAs tend to concentrate.

**Figure 20:**
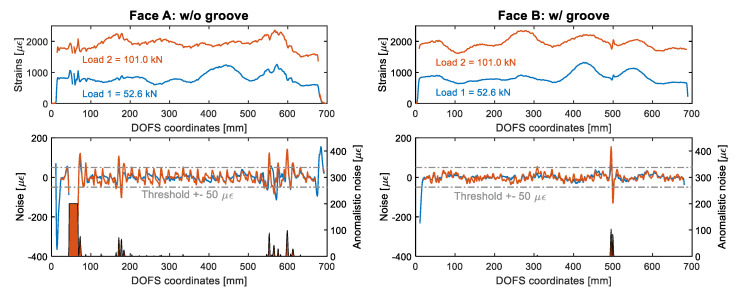
Member 8D16’s strain profiles (**top**) and noise level (**bottom**) study for strain reading anomalies (SRA) analysis.

Whenever data is absent it can also be considered an anomaly as the viewer is also missing out on relevant information just like with an SRA. Except that, whilst with small SRAs a trend can still be distinguishable, with absent data no such thing can be done. Therefore, the used SRA detection code was tweaked to attribute an anomalistic noise of 200 με to any DOFS section with missing data (as visible in Figure 20).

**Figure 21:**
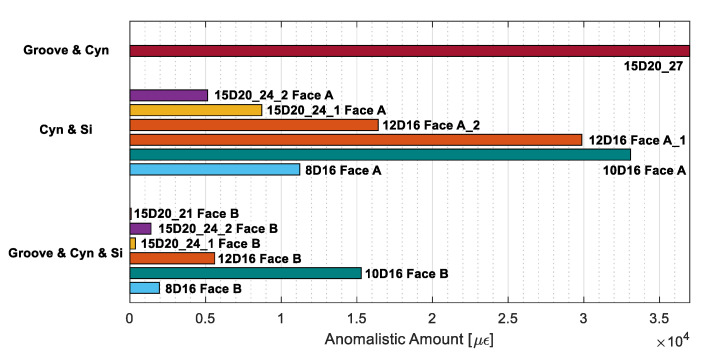
Total amount of anomalistic readings grouped per bonding technique used.

With these observations in mind a final calculation of the total amount of SRAs can be performed per every bonding technique. Its outcome, represented in Figure 21, shows a clear advantage of the groove, CYN and SI technique as it averagely welcomes a distinctly smaller amount of SRAs compared to the other two.

## 6. Conclusions

The present article provided a contribution to the effort of establishing the best DOFS/rebar bonding technique whenever the instrumented bar is to be placed on the inside of RC structures. To this end, a first step was taken by looking into the state of the art on the bonding techniques used so far by several researchers. Despite some scattered data was provided, no clear indication could be extracted on the most performant technique to both glue and protect the DOFS. Thus, the experimental campaign, core subject of the paper, took on itself the challenge of testing multiple bonding techniques on multiple rebars, non-cracking and cracking RC ties tested in tension in order to check their performance.

The following conclusion can be extracted:

- The presence of a longitudinally incised groove and silicone (SI) as extra protection for the DOFS against external factors is un-influential on bare rebars subjected to pure tension or bending;
- The combination of a groove, cyanoacrylate (CYN) and SI is the bonding technique that leads to the smallest increase of noise magnitude of the DOFS profiles with increasing load;
- None of the studied bonding techniques could completely guarantee the removal of all SRAs. Yet, the combination of a longitudinally incised groove, CYN and SI is the bonding technique that leads to the smallest amount of strain reading anomalies (SRAs) thus preserving the readability and reliability of the strain data;
- An unexpected phenomenon, possibly connected to the under-performance of the above bonding technique, causes an alteration of the DOFS strain readings whenever the fiber is in the presence of extra bending-induced tension (smoothened strain profiles as in Figure 16, Face A) and/or monitoring strains in cracked cross-sections (magnifying their amount as in Figure 18);
- Despite its superior performance compared with other bonding techniques, the combination of groove, CYN and SI suffers gravely of the above issue and thus cannot be referred to as an ideal DOFS/rebar bonding technique but simply as the most performant among the modern-day ones;
- Further research is then required on substituting SI with an equally protective adhesive that does not transfer any internal extra stretches to the DOFS, allowing it to sample strictly the rebar’s strains. Furthermore, the bonding performances should be studied with different testing conditions such as dynamic (cyclic) and impact testing.

## Figures and Tables

**Table 1:** Slope coefficient of the average strain reading noise growth with load for the presented RC ties and subdivided per bonding technology and presence/absence of concrete.

	Groove and CYN		CYN and SI		Groove and CYN and SI	
Inside Concrete:	No	Yes	No	Yes	No	Yes
15D20_27	0.2539	∞	-	-	-	-
15D20_21	-	-	-	-	0.2084	0.1538
15D20_24_1	-	-	A: 0.2298 B: 0.1815	A: 0.3636 -	- B: 0.1041	- B: 0.0360
15D20_24_2	-	-	A: 0.3247 B: 0.0906	A: 0.2015 -	- B: 0.0698	- B: 0.0531
